# Experimental Studies on the Influence of Plasma Treatment of Polyethylene in Carbon Fiber Composites: Mechanical and Morphological Studies

**DOI:** 10.3390/polym14061095

**Published:** 2022-03-09

**Authors:** Sumesh K R, Jakub Anton, Petr Spatenka, Hana Jelinek Sourkova

**Affiliations:** Department of Materials Engineering, Faculty of Mechanical Engineering, Czech Technical University in Prague, Karlovo Namesti 13, CZ-12135 Prague, Czech Republic; jakub.anton@fs.cvut.cz (J.A.); petr.spatenka@fs.cvut.cz (P.S.); hana.sourkova@fs.cvut.cz (H.J.S.)

**Keywords:** carbon fiber, polyethylene, plasma treatment, mechanical properties

## Abstract

This research focused on enhancement of mechanical properties in carbon fiber (CF)-filler-reinforced linear low-density polyethylene (PE) matrix composites. A hand layup method using an oven was used as the fabrication method. Improvement in adhesion was achieved by oxygen plasma treatment to the PE matrix. CF and PE were initially mixed by normal stirring, ultrasonication and mechanical stirring before being filtered and dried for fabrication. Better tensile results were observed with a plasma-treated polyethylene (PEP)/10 wt.% CF combination, with a maximum tensile strength of 21.5 MPa and improvement in the properties of up to 12.57% compared to non-plasma PE with the same CF addition. The addition of carbon fibers at 13 and 15 wt.% resulted in a reduction in the tensile strength properties to 18.2 MPa and 17.7 MPa, respectively. This reduction in tensile strength was due to agglomeration of CF with plasma- and non-plasma-treated PE. The fabrication condition of 180 °C temperature for 20 min showed better tensile properties than other conditions. The SEM results following tensile testing revealed enhanced CF filler adherence with plasma PE results, as well as fewer surface deformations. A higher flexural strength of 25.87 MPa was observed for the plasma treated PE/7 wt.% CF combination.

## 1. Introduction

Polyethylene is one of the most effective polymers used in various fields for thin films, adhesives, medical implants, electronics, battery and orthopedic applications [1,2]. It is usually fabricated using rotomolding for industrial applications. The polyethylene (PE) matrices are mixed with various fillers and reinforcements to enhance applications. Fiber and matrix adhesion are vital factors in determining the mechanical properties of the composites. These can be improved by plasma treatment of polyethylene matrices, which significantly enhances compatibility between fibers and matrices [2,3,4]. This method is an eco-friendly approach creating a wide range of reactive species in the polymers, such as amine, hydroxyl, carboxyl, ether, peroxide and carbonyl groups, depending on the surrounding medium. Plasma treatment enhances the surface roughness and hardness properties of the composites [5,6,7]. 

A sandwich layer of polyethylene and polyamide (PA) matrices of two different polarities results in better bonding with plasma treatment using oxygen. A peel strength of 7.657 N/mm was observed which was twice that of untreated samples. Delamination was observed in untreated PE samples. A flexural strength of 35.4 MPa was observed with PE and PE with plasma treatment, far higher than an untreated combination [8]. Plasma-treated PE showed better tensile, flexural and impact properties while incorporating natural reinforcements. The functional groups in the treated PE directly interact with the hydroxyl groups in lignocellulosic coir fibers improving the adhesion of the composites [9]. Glass fiber (GF)-based PE composites showed optimum tensile strength at 10 wt.% GF with plasma PE. Best results for the tensile modulus were observed for plasma-treated PE with 20 wt.% GF [10]. 

Carbon fiber is one of the most useful materials used in automotive, sports and aerospace applications due to its high corrosion resistance, mechanical properties, and excellent coefficient of thermal expansion [11,12]. The incorporation of carbon fiber up to 20 wt.% in polypropylene (PP)/high-density polyethylene (HDPE) composites showed 60%, 18%, and 23% enhancement in impact, tensile and flexural properties of matrices, respectively [13]. Agglomeration is a major factor that reduces the compatibility of carbon fiber with resins at higher fiber substitution, resulting in reduced overall mechanical properties of the composites [12]. The incorporation of carbon fiber at 80 wt.% produced a maximum flexural strength of 64.37 MPa and higher hardness compared to other combinations of epoxy resin [14]. Mixing of carbon fibers up to 25 wt.% in HDPE increased tensile strength and the tensile modulus of the combination. The spray-coating of carbon nanotube (CNT) at 1 wt.% further enhanced the tensile strength and tensile modulus properties of carbon fiber/HDPE-based composites [15]. Recycled carbon fibers have similar applications in automobiles and other industrial applications, with lower cost in comparison to normal carbon fibers, but recycled carbon fibers lack the mechanical properties of polymer matrices due to the effects of recycling [16,17,18]. 

Oxygen plasma treatment at 80 W to carbon fibers slightly enhanced their tensile properties. Good interfacial shear strength of 71 MPa was observed for 20 W plasma treatment [19]. The shape memory of polymers of an epoxy-based matrix resulted in improved mechanical properties with incorporation of continuous and short carbon fibers into the combination [20]. Carbon reinforced materials, such as carbon fibers, single and multi-wall carbon nanotubes and carbon black (CB), have also been used for electrical conductivity applications with various thermo- and thermo-setting plastics. Carbon black and carbon nanotube fillers reduced the mechanical properties of polyethylene-based composites [21,22,23].

The plasma treatment of polycarbonate/carbon fiber/carbon nanotubes enhanced the dynamic mechanical properties of the combination [24]. Radiofrequency plasma treatment, using air, argon and a combination of air and argon, for both PEEK and carbon fibers, increased surface roughness and provided good mechanical interlocking with CF and PEEK. This enhanced the interfacial strength of the PEEK composites [25]. The combination of glass/polypropylene, pure polypropylene and HDPE composites showed improved interfacial bonding having good surface energy with oxygen plasma treatment [26]. 

The literature suggests that plasma treatment is an effective technique in improving the interaction between matrix and fiber phase. It is also known that recycled carbon fiber is a good alternative to conventional carbon fiber due to its lower cost, environmentally friendly characteristics and comparable mechanical properties. More work in this field is required to bring recycled carbon fibers to a large-scale market. There is no reported research relating to plasma-treated polyethylene and carbon fibers which addresses tensile strength, tensile modulus, flexural strength, flexural modulus and morphological properties. In this study, the influence of plasma treatment of PE was characterized using FTIR and XPS analyses. Different fabrication conditions were applied to determine the optimal conditions for oven processing. Carbon fibers were treated with plasma in the final stage for confirmation.

## 2. Materials and Methods

### 2.1. Materials

Polymer powder of hexane-based linear low-density polyethylene (PE) of Dowlex^TM^ 2629.10UE purchased from Dow Corporate was used as the matrix material. This polymer has a melt flow index of 3.8 g/m (ASTM D1238), density of 0.9370 g/cm^3^ (ASTM D792), and bulk density of 0.346 g/cm^3^ (ASTM D1895). The polymer has been used for the manufacture of bulk and freezer containers, especially drums used for chemicals [3]. There was no pre-treatment provided before the plasma treatment of polyethylene and carbon fibers. The plasma treatment was applied to the polyethylene powder (PEP) using an industrial purpose device (LA 400) for surface treatment (a. s. Turnov, Czech Republic). The plasma was applied using two microwaves with a total power of 2 kW. In oxygen plasma treatment, a gas flow of 100 sccm was applied with a treatment time of 30 s. The procedure for plasma treatment was performed as previously described [3].

Recycled carbon fibers prepared from PAN fibers with a purity of 99% and size of 80–350 µm were used as the filler for the PE matrix. The recycled carbon fibers were supplied by MSV STUDENKA sro, Bilovec, Czech Republic. The fiber length was 8 mm, with diameter of 7 µm, density of 1.8 g/cm^3^, and bulk density of 250–550 g/L. The fibers have high strength and flexibility, low density, corrosion, heat resistance, abrasion resistance and electrical conductivity applications. The carbon fibers were treated using an industrial purpose device (LA 400) having oxygen as the working gas with a flow of 100 sccm.

### 2.2. Fabrication

**Figure 1:**
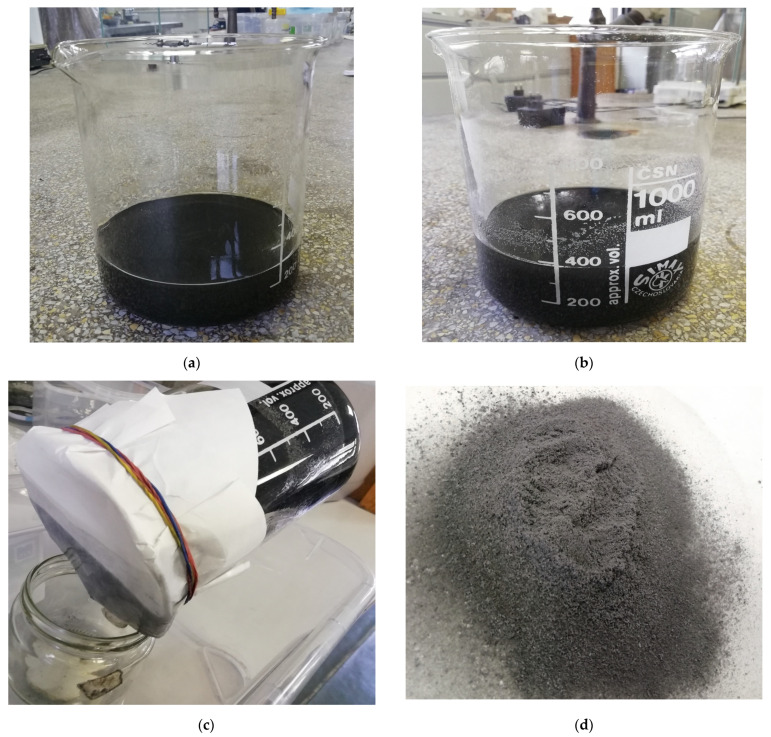

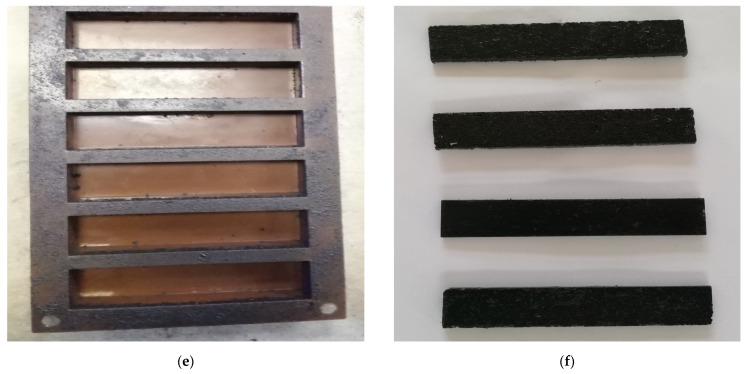
Fabrication procedures for CF/LLDPE composites: (**a**) CF/IPA mixture; (**b**) CF/IPA/PE mixture after ultrasonication; (**c**) filtration of CF/IPA/PE mixture, (**d**) final dried powder for fabrication, (**e**) silicon mold for fabrication and (**f**) CF/PE composites after fabrication process.

A hand layup method was used in the development of carbon fiber/linear low-density polyethylene (PE)-based composites. The carbon fiber (CF) was reinforced at 3, 5, 7, 10, 13 and 15 wt.% in the PE matrix. Carbon fibers were initially combined with isopropyl alcohol (IPA) during the mixing process (Figure 1a). Manual stirring followed by ultrasonication (50 °C, 40 kHz for 60 min) (Figure 1b) and mechanical stirring (for 10 min) was carried out for proper mixing of the PE matrix, IPA and carbon fiber. Finally, the wet mixture was filtered using filter paper (Figure 1c) to remove isopropyl alcohol, and dried using a hot oven in atmospheric air. The dried powders (Figure 1d) were sifted to reduce the residual content of mixtures and placed in silicon molds of dimensions 100 × 25 × 3 mm^3^ (Figure 1e). The samples were rolled using a roller to produce a finely finished composite specimen, then kept in a hot oven at 180 °C for 20 min and allowed to dry at room temperature (Figure 1f). Similar procedures were followed for the plasma-treated PE and the plasma-treated CF. Different fabrication conditions between 180 and 200 °C temperature and time interval of 20–60 min were tested to optimize fabrication. Plasma-treated PE was used to improve adhesion in the matrix/fiber phase.

### 2.3. Testing Methods

Tensile testing (ISO 527) was carried out using a universal testing machine (UTM) with gauge length of 6 cm and speed of 5 mm/min. All the mechanical results were obtained with an average of 10 samples. The same machine was used in three-point bending testing to assess flexural properties using an ASTM D 790 standard machine with a speed of 2 mm/min. After tensile testing, samples were tested using SEM [JSM-7600F (JEOL, JP)] to determine the adhesion and the distribution of carbon fibers in the PE matrix. Sputtering of the chromium layer was performed to enable proper passage of electron beams into the specimen without charging. The combinations were evaluated at a voltage and working distance of 1–5 kV and 5.4–10 mm, respectively.

## 3. Results

### 3.1. Tensile Testing

**Figure 2:**
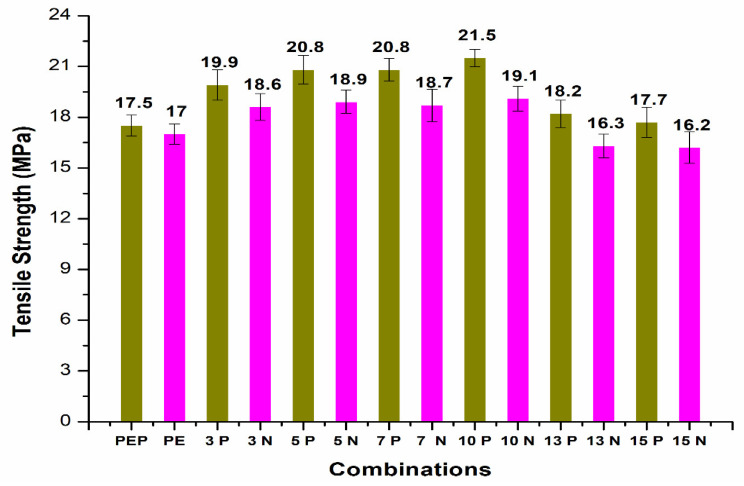
Tensile strength of CF/PE composites.

**Figure 3:**
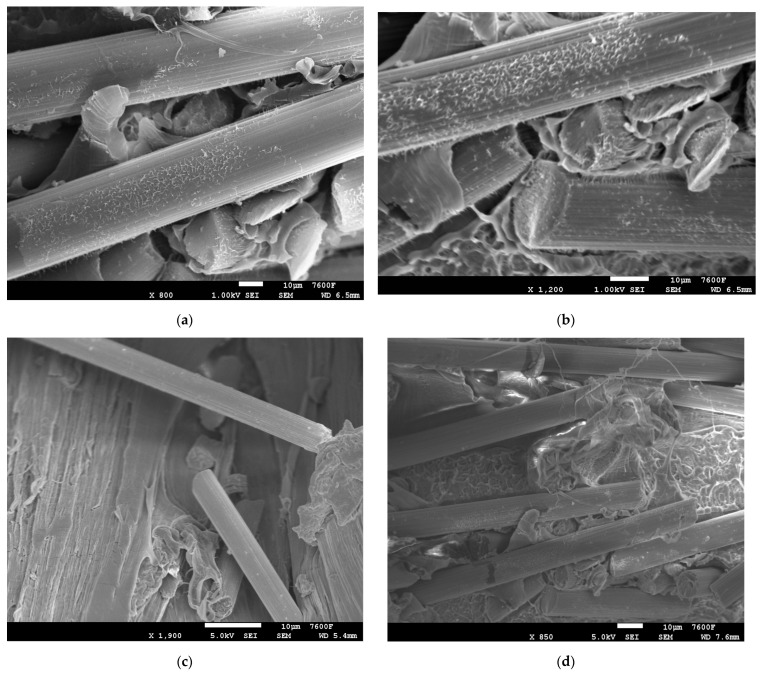
SEM images: (**a**,**b**) 10% CF/plasma PE, (**c**) 5% CF/plasma PE and (**d**) 15% CF/plasma PE combinations.

Tensile testing using carbon fiber (CF)/polyethylene (PE) composites clearly showed enhancement of tensile strength with plasma treatment of PE (Figure 2). For the initial combination with plasma treatment without carbon fiber, the tensile results showed a smaller increase in the tensile properties of 2.94%. In Figure 2, PEP and PE are the plasma treated and non-plasma-treated PE, respectively. 3P and 3N are the 3 wt.% CF with plasma-treated PE and non-plasma-treated PE, respectively. Similarly, 5P, 5N, 7P, 7N, 10P, 10N are the 5 wt.% CF, 7 wt.% CF, 10 wt.% CF with plasma and non-plasma treated PE, respectively. The other combinations (13P, 13N, 15P, and 15N) follow the same principle. In previous studies, it was shown that oxygen-plasma-treated Dowlex^TM^ 2629.10UE had good wettability, which enhanced the adhesion of matrix with fillers [3,8]. It was evident from the SEM results shown in Figure 3 that the plasma treatment of PE enhanced the adhesion behavior of non-polar polyethylene polymer by providing better chemical interaction and mechanical interlock in the matrix. Plasma treatment enables addition of the active species, which creates better surface roughness for enhancing the adhesiveness of the PE matrix [4,5,9]. The 10% CF (Figure 3a,b) combination with plasma-treated PE showed good distribution of carbon fibers in the PE matrix with fewer surface deformations, thus exhibiting good mechanical properties. There was good interfacial adhesion with the PE matrix and 5% CF filler resulting in good properties (Figure 3c). The 15% CF (Figure 3d) material showed slightly more deformations in the matrix surface due to high CF incorporation in the PE matrix. Surface deformations negatively affected the mechanical properties of the polymer-based composites [13].

**Figure 4:**
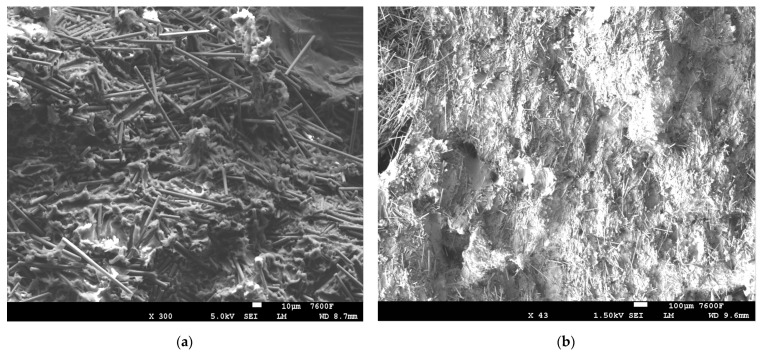
SEM images: (**a**) 5% CF/Plasma PE and (**b**) 10% CF/plasma PE combinations.

The plasma-treated PE effect was much more evident with incorporation of carbon fiber filler. Initial addition of carbon fibers (CF) at 3 wt.% increased tensile strength up to 19.9 MPa, which was higher than for the non-plasma-treated PE with an 18.6 MPa tensile strength value. Improvement in the tensile strength with plasma treatment increased up to 6.99%. In the combinations with 5 wt.% and 7 wt.%, CF tensile strength reached 20.8 MPa, with increases in strength up to 10.05% and 11.23%, respectively. A clear trend in the improvement of tensile strength with addition of carbon fiber with plasma-treated PE was observed in consequence. Interfacial adhesion between the matrix and carbon fibers by the plasma-treated PE was the main reason behind the observed enhancement. The overall distribution of 5% CF and 10% CF in plasma PE is clearly visible in Figure 4.

The oxygen plasma treatment activated the surface as etching occurred, which was the main mechanism of the treatment. In previous studies with Dowlex^TM^ 2629.10UE, it was evident that slight plasma treatment of oxygen created significant increase in the wettability nature of polyethylene composites. This demonstrates that polymer powder does not require more oxygen groups to achieve wettability. The shorter plasma treatment provided low pressure oxygen and enabled formation of polar groups due to the plasma. Longer exposure induced additional abrasion on the PE composites, resulting in rougher surfaces. The oxygen plasma treatment enabled low-surface-energy modification of the polymers, which improved adhesion for better application [3,4,5,6].

**Figure 5:**
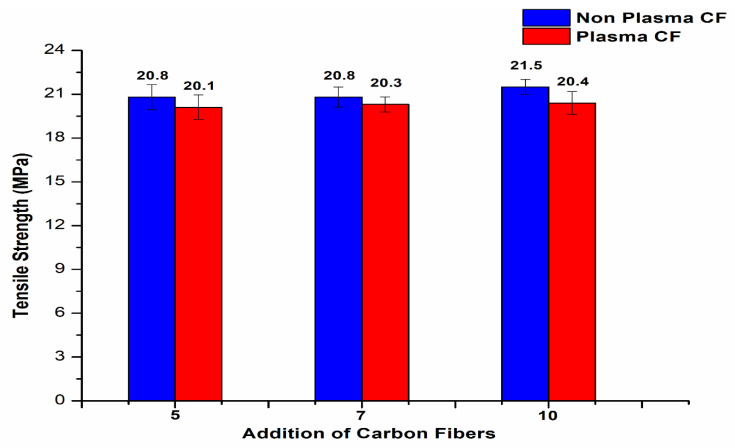
Tensile strength of CF/plasma PE composites.

The best results were observed with 10 wt.% CF, with a maximum tensile strength of 21.5 MPa and improvement in the properties up to 12.57% compared to non-plasma PE with the same CF addition. The addition of carbon fibers at 13 and 15 wt.% showed reduction in the tensile strength properties to 18.2 MPa and 17.7 MPa, respectively. The reduced tensile strength was due to agglomeration of CF with plasma- and non-plasma-treated PE. The incorporation of plasma-treated CF with plasma PE did not result in any further improvement in the tensile strength of the PE composites (Figure 5). It can be clearly seen that plasma treatment of CF resulted in reduction in the tensile strength to 20.1 MPa, 20.3 MPa and 20.4 MPa with addition of 5, 7 and 10 wt.% CF, respectively

**Figure 6:**
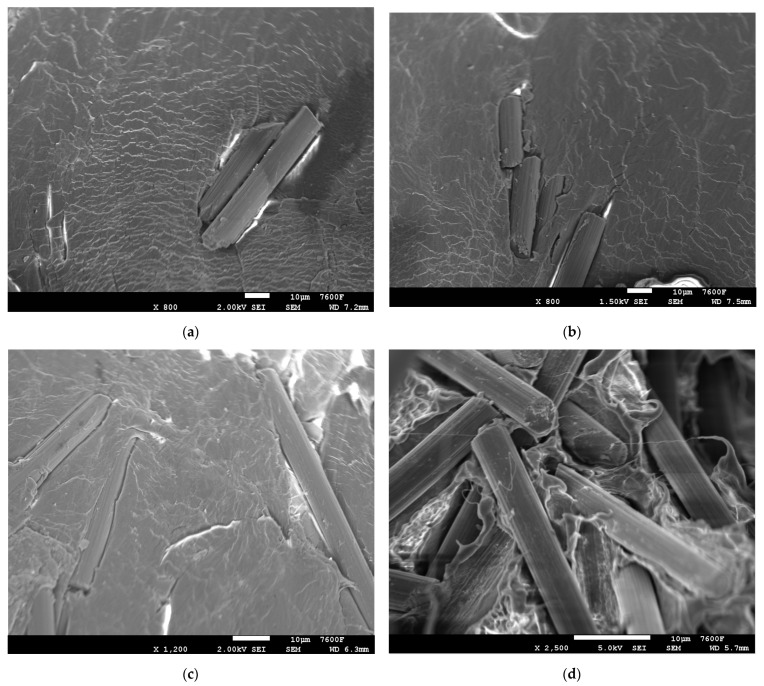
SEM images: (**a**,**b**) 5% CF/PE, (**c**) 10% CF/PE and (**d**) 15% CF/PE combinations.

**Figure 7:**
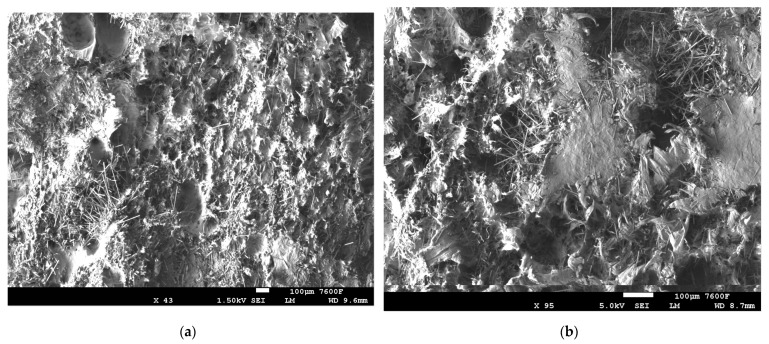
SEM images: (**a**) 10% CF/PE and (**b**) 15% CF/PE combinations.

The SEM images in Figure 6 clearly indicates the surface deformations in the combinations without plasma-treated PE after mechanical testing [9]. The 5% CF/PE, as shown in Figure 6a,b, caused the fiber and matrix breakages in the composites which negatively affect the overall properties of the combination. The gap or void space between the PE matrix and the CF fiber is clearly visible in Figure 6c, which negatively affected the properties. Similar deformations can be seen in Figure 6d with matrix breakages and fiber breakages in the combinations. The increased addition of CF at 15%, creating an agglomeration effect, also resulted in surface deformations. The distribution of 10% CF and 15% CF in the PE matrix clearly increased deformation in the surface, as shown in Figure 7.

**Figure 8:**
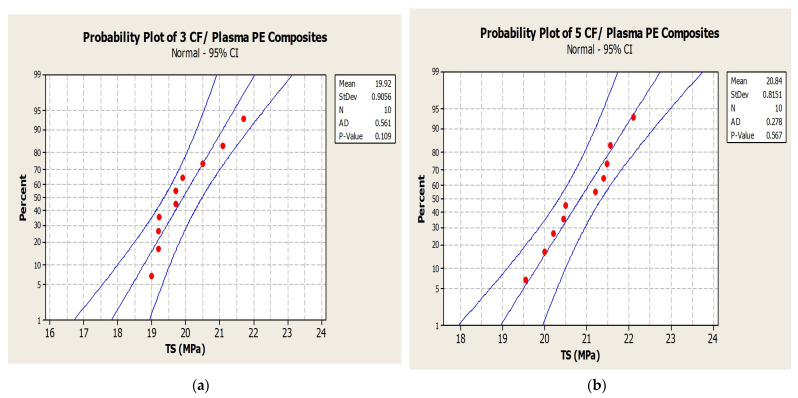

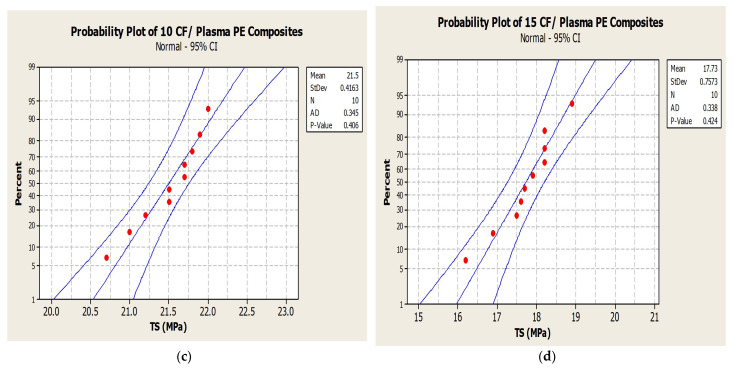
Normal probability plot for tensile strength: (**a**) 3% CF/plasma PE, (**b**) 5% CF/plasma PE, (**c**) 10% CF/plasma PE and (**d**) 15% CF/plasma PE combinations.

**Figure 9:**
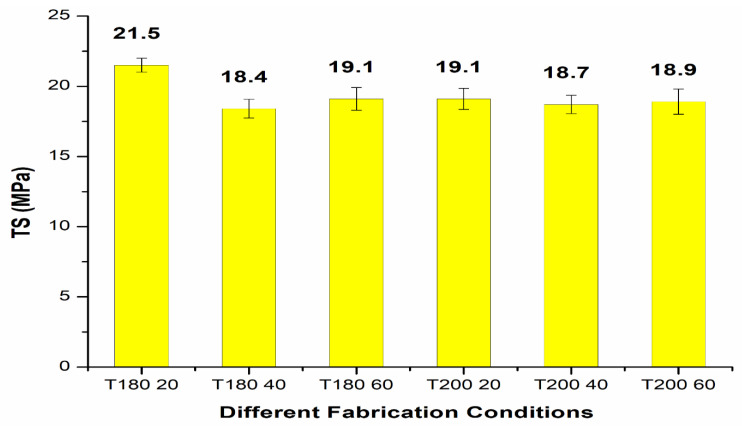
Tensile strength (TS) of 10 wt.% CF/plasma PE under different fabrication conditions.

The normal probability plot (Figure 8) chart of tensile strength (TS) for 3, 5, 10 and 15 wt.% CF with plasma PE shows readings well within the specified limit; the distribution, standard deviation and mean values are shown in the figure. The results for tensile strength with CF/PE showed better tensile properties for the 10 wt.% CF/plasma-treated PE combination with a tensile strength value of 21.5 MPa. The same composite combination was tested under different fabrication conditions (Figure 9). Initial test was performed with ‘T 180 20’, i.e., 180 °C temperature for 20 min. Similarly, T 180 40, T 180 60, T 200 20, T 200 40, and T200 60 were fabrication conditions at different temperatures and with different time intervals. Apart from the T180 20 fabrication condition, all other conditions showed reduced properties.

**Figure 10:**
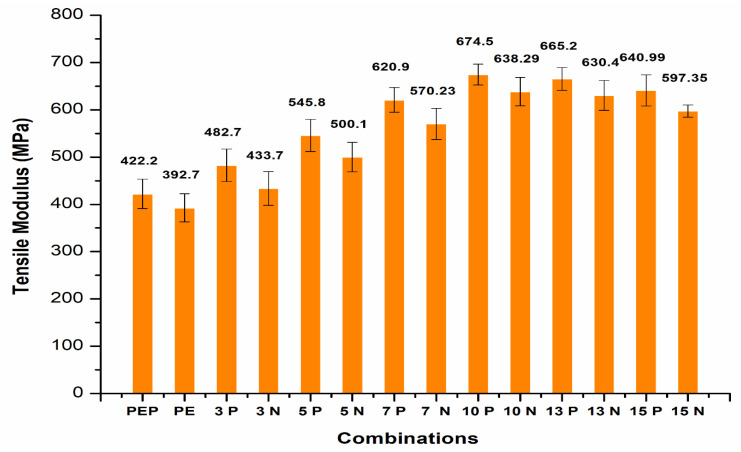
Tensile modulus of CF/PE composites.

The tensile modulus properties of the CF/PE composites (Figure 10) showed strong improvement with plasma treatment of PE and with carbon fiber addition. The incorporation of plasma-treated PE with 3, 5, 7 and 10 wt.% carbon fibers resulted in tensile modulus properties of 482.7 MPa, 545.8 MPa, 620.9 MPa, and 674.5 MPa, respectively. The properties were higher for non-plasma-treated PE with the same carbon fiber addition. Similar to tensile strength, tensile modulus also showed better properties at 10 wt.% CF with plasma PE. Further addition in between 13–15 wt.% CF was associated with reduced tensile modulus properties.

### 3.2. Flexural Testing

**Figure 11:**
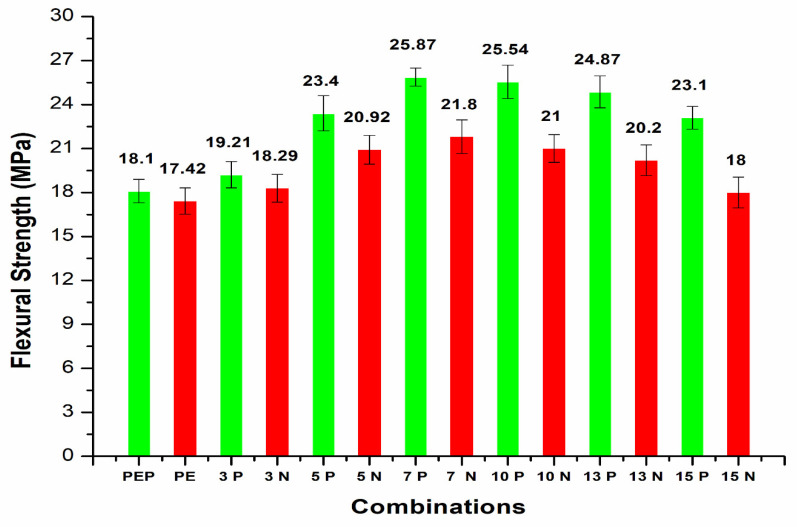
Flexural strength of CF/PE composites.

The flexural strength (FS) of CF/PE composites (Figure 11) followed the same pattern as the tensile characteristics, with plasma-treated PE and carbon fiber incorporation increasing flexural strength. It can be clearly seen that plasma treatment to PE resulted in slight improvement of flexural strength from 17.42–18.1 MPa for the initial combination.

The addition of carbon fibers (3 wt.%) with plasma polyethylene powder resulted in enhancement from 18.29 to 19.21 MPa in the flexural properties. Similar enhancement in the flexural properties was observed for 5, 7, 10 wt.% with the plasma-treated PE-incorporated carbon fibers. A maximum flexural strength of 25.87 MPa was observed with the plasma-treated PE/7 wt.% CF combination. 

**Figure 12:**
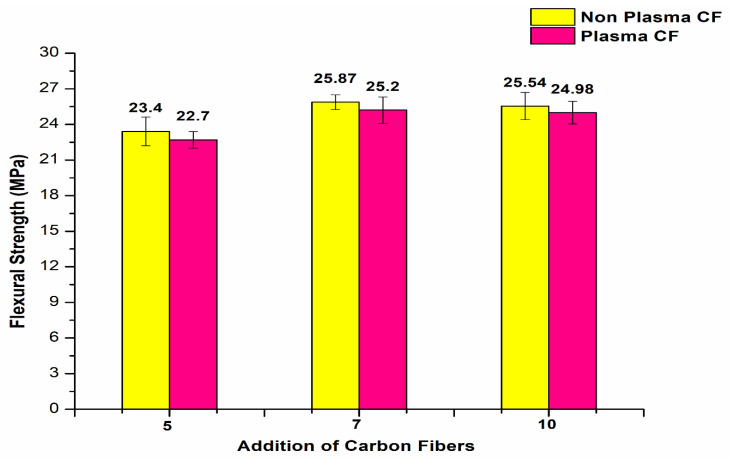
Flexural strength of CF/plasma PE composites.

**Figure 13:**
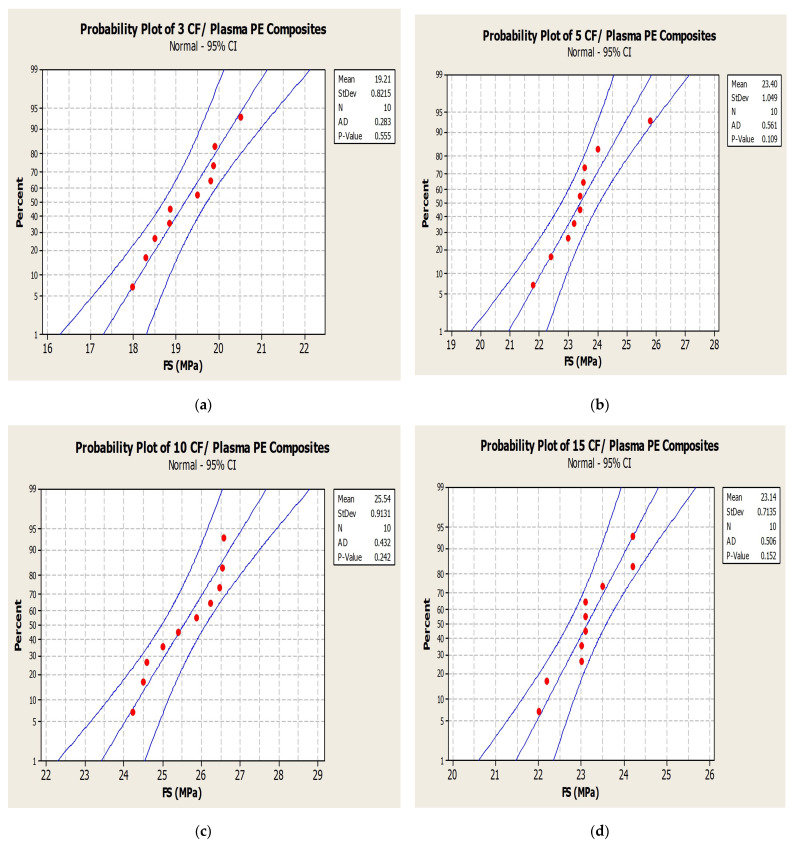
Normal probability plot for flexural strength: (**a**) 3% CF/plasma PE, (**b**) 5% CF/plasma PE, (**c**) 10% CF/plasma PE and (**d**) 15% CF/plasma PE combinations.

**Figure 14:**
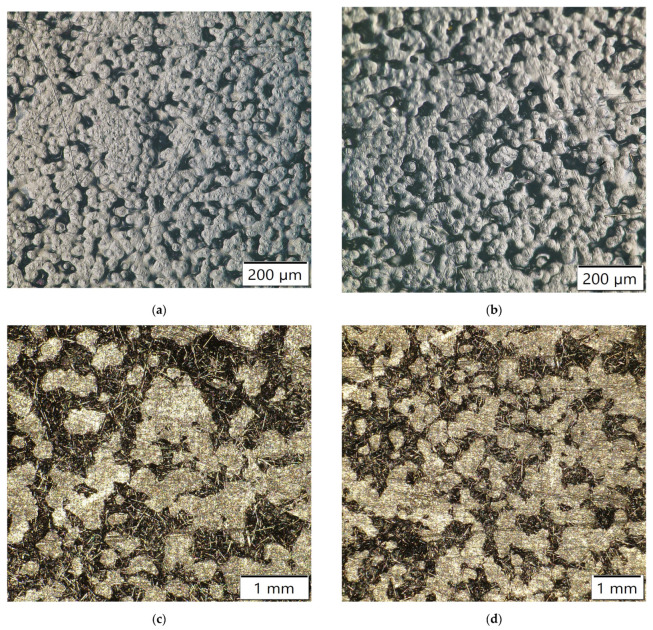
Surface images using optical microscopy: (**a**) PE, (**b**) plasma PE, (**c**) 7% CF/non-plasma PE and (**d**) 7% CF/plasma PE combinations.

**Figure 15:**
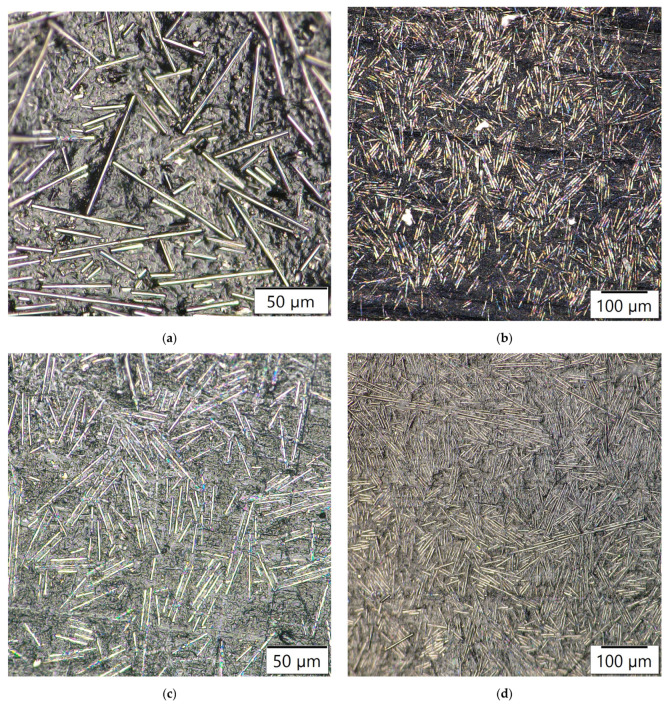
Surface images using optical microscopy: (**a**,**b**) 10% CF/non-plasma PE and (**c**,**d**) 10% CF/plasma PE combinations.

The addition, 10 wt.% CF/plasma-treated PE showed almost similar flexural properties at 25.54 MPa. Further addition of CF with plasma-treated PE resulted in reduced flexural strength for 13 and 15 wt.% to 24.87 MPa and 23.1 MPa, respectively. Plasma treatment of oxygen was associated with the presence of polar groups, such as hydroxyl, carbonyl and carboxyl groups. The presence of these polar groups induced creation of polymer chains with free radicals, making it possible to add or interlock functional groups to the surface of the polymers. This would have had a favorable impact on the polymer surface activation by increasing the adhesive nature of the composites [3,8]. Agglomeration of carbon fibers resulted in reduction in the flexural properties of the polymer composites [12]. The 13 and 15 wt.% CF showed improvement in the properties by incorporation of plasma-treated PE compared to untreated PE with the same carbon fiber addition. The plasma treatment of 5, 7 and 10 wt.% CF did not show any further improvement in the flexural strength of the plasma-treated PE based composites (Figure 12). The flexural properties were reduced from 23.4 to 22.7 MPa, 25.87 to 25.2 MPa and 25.54 to 24.98 MPa in plasma-treated 5, 7 and 10 wt.% CF/plasma PE, respectively, compared to the same CF combination without plasma/plasma PE combination. Interfacial interaction between filler and matrix were reduced, associated with a decline in the flexural strength of the combination. The normal probability plot (Figure 13) of flexural strength (FS) in 3, 5, 10 and 15 wt.% CF with plasma PE showed readings well within the specified limit; the distribution, standard deviation and mean values are shown in the figure itself. Surface images using optical microscopy (Figure 14 and Figure 15) showed evenly dispersed CF for the plasma-treated PE in 7% CF (Figure 14d) and 10% CF (Figure 15c,d). This improved the overall mechanical properties of the PE matrix. It can also be seen from the images that plasma treatment in PE enhanced the adhesion in the fiber/matrix phase with less void space, adding to the tensile and flexural properties of the polymer composites [9,10]. 

**Figure 16:**
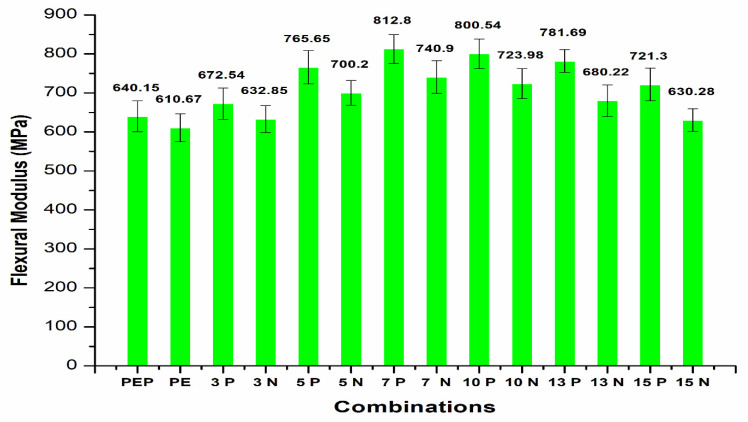
Flexural modulus of CF/PE composites.

**Figure 17:**
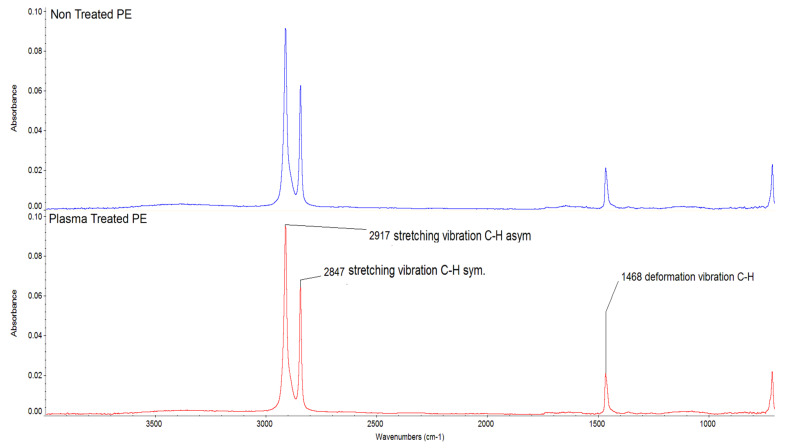
FTIR results for non-treated and plasma-treated PE samples.

**Figure 18:**
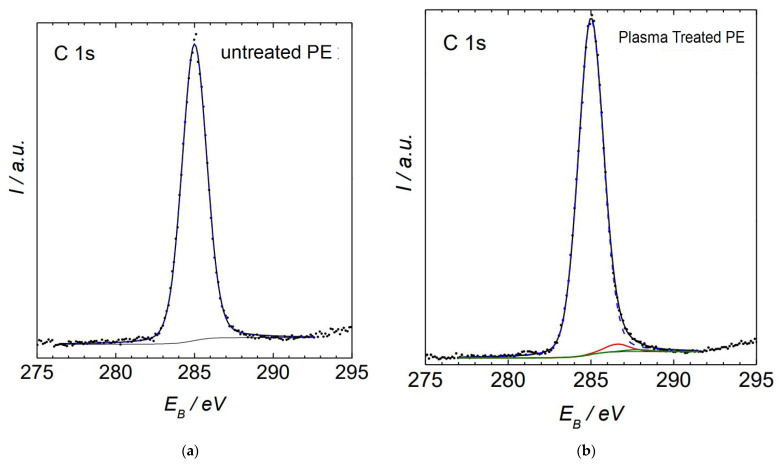
XPS results of PE powder: (**a**) untreated PE [27], (**b**) plasma-treated PE. Black line with O-C=O bond, blue line with C-C bond, green and red line with C=O and C-O bonds, respectively.

The flexural modulus results (Figure 16) for the CF/PE composites showed a trend in enhancement with plasma treatment of PE for the CF-based composites. It can be clearly seen that the flexural modulus properties increased with plasma-treated PE incorporating CF compared to the pure PE matrix with the same carbon fiber addition. The CF filler also strongly enhanced the mechanical properties of the PE composites. The maximum flexural modulus properties were observed with 7 wt.% CF/plasma PE with a value of 812.8 MPa. The FTIR results (Figure 17) for the treated and non-treated PE samples did not show any changes in the functional groups. Both PE samples showed peaks at 2917cm^−1^ and 2847 cm^−1^ due to stretching vibration of C-H asymmetric and C-H symmetric groups. There was also a peak at 1468 cm^−1^ due to deformation vibration of C-H groups [6]. The XPS results showed (Figure 18) concentration of plasma-treated PE of 30 s for O-C=O groups to be much less, making it hard to be certain. The percentage of concentration observed for C-O groups was 2.2% and for C=O groups was 0.6%. Similar functional groups with plasma-treated PE were observed in our recent paper [27]. In the plasma-treatment process of PE, chemical interaction of one O_2_ atom with the polymer surface produced -OH groups and functional groups with richer O_2_ [3].

## 4. Conclusions

- The mechanical properties of carbon fiber (CF)/polyethylene (PE) composites showed better tensile and flexural properties with plasma-treated PE and CF incorporation.
- The best tensile results were observed with 10 wt.% CF, with a maximum tensile strength of 21.5 MPa and improvement in the properties up to 12.57% compared to non-plasma PE with the same CF addition.
- The addition of carbon fibers at 13 and 15 wt.% resulted in reduction in the tensile strength properties to 18.2 MPa and 17.7 MPa, respectively. This reduction in tensile strength was due to agglomeration of CF with plasma- and non-plasma-treated PE.
- The fabrication condition of 180 °C temperature for 20 min in an oven showed optimized tensile properties compared to other conditions.
- The SEM results after tensile testing showed improved adhesion of CF filler with plasma PE, resulting in fewer deformations in the surface.
- The incorporation of plasma-treated PE with 3, 5, 7 and 10 wt.% carbon fibers resulted in tensile modulus properties of 482.7 MPa, 545.8 MPa, 620.9 MPa, and 674.5 MPa, respectively. These values were higher than for non-plasma-treated PE with the same carbon fiber addition.
- Similar to tensile strength, the tensile modulus also showed better properties at 10 wt.% CF with plasma PE.
- The addition of carbon fibers (3 wt.%) with plasma polyethylene powder resulted in enhancement from 18.29 to 19.21 MPa in the flexural properties. Similar enhancements in the flexural properties were observed in 5, 7, 10 wt.% with the plasma-treated PE incorporating carbon fibers.
- A maximum flexural strength of 25.87 MPa was observed with the plasma-treated PE/7 wt.% CF combination. The addition of 10 wt.% CF/plasma-treated PE showed almost identical flexural properties with a flexural strength value of 25.54 MPa.
- Further addition of CF with plasma PE resulted in decline in flexural strength in 13 and 15 wt.% with flexural strength values of 24.87 MPa and 23.1 MPa, respectively.
- The combination of 7 wt.% CF/plasma PE resulted in a maximum flexural modulus value of 812.8 MPa.
- In terms of tensile and flexural properties, plasma-treatment of oxygen with CF did not appear to be effective with polyethylene composites.
- These combinations can be used for small scale applications in the automobile industry. In future research, electrical conductivity properties can also be tested.

## Data Availability

The data presented in this study are available on request from the corresponding author.
